# Supplementary material for: Video meeting signals: Experimental evidence for a technique to improve the experience of video conferencing
Source: PLoS One. 2022 Aug 3;17(8):e0270399. doi: 10.1371/journal.pone.0270399 (PMC9348663; doi:10.1371/journal.pone.0270399)
Supplement: S1 File — (PDF) [file pone.0270399.s001.pdf]

SUPPLEMENTARY MATERIALS
Video Meeting Signals
A randomised controlled trial of a technique to improve the experience of video conferencing

Paul Hills, Mackenzie Clavin, Miles R. A. Tufft & Daniel C. Richardson
Department of Experimental Psychology, University College London

S1 Table. Experiment 1 Post seminar survey items

THEME	ITEM INDICIES	(*REVERSED SCORED)
PERSONAL EXPERIENCE	1.	At the end of the seminar I experienced 'zoom fatigue'*
	2.	I enjoyed the seminar
	3.	I felt positive at the end of the seminar
	4.	I was active
	5.	I was bored in the seminar*
	6.	The seminar was engaging
OUTCOMES	1.	I now have a better understanding of the assigned paper today
	2.	We considered lots of different ideas before deciding on our hypothesis
	3.	It was easy to find a consensus
	4.	I contributed towards the hypothesis that the group chose
	5.	I am more likely to use the paper in a essay or exam answer
	6.	The seminar had a positive impact on my learning
	7.	I am satisfied with the hypothesis chosen by my group
	8.	The seminar was interactive
	9.	The seminar was productive
	10.	I found the seminar useful
	11.	It was time well spent
	12.	I heard a variety of opinions about the paper
MECHANICS	1.	I was able to share my thoughts and opinions
	2.	It was easy to exchange ideas
	3.	We often went off topic*
	4.	I found it hard to speak *
	5.	I was often interrupted *
	6.	I feel my voice was heard
	7.	There was open communication
	8.	The seminar was well structured
	9.	The seminar leader was an effective facilitator
GROUP AFFILIATION	1.	Everyone had their voice heard in seminars
	2.	My seminar group was motivated
	3.	I know the people my seminar group well
	4.	Which of these pictures represents your relationship to the other students after your last meeting?<br>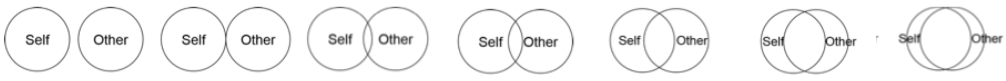
	5.	My opinion was valued by the group
	6.	People ideas were respected
	7.	I would like to work with this group in the future

Experiment 1. Bayesian model – Survey results

Our model was specified as

response ~ training * week * theme + (1 | seminar group) + (1 | participant)

The model's priors were set as follows:

~ normal (location = (0, 0, 0, 0, 0, 0, 0, 0, 0, 0, 0, 0, 0, 0, 0),
scale = (2.49, 2.49, 2.49, 2.49, 2.49, 2.49, 2.49, 2.49, 2.49, 2.49, 2.49, 2.49, 2.49, 2.49, 2.49))

We ran a Bayesian MCMC (link = logit) model (4 chains, each with iter = 2000; warmup = 1000; thin = 1; post-warmup = 1000) using the rstanarm package (v. 2.18). The model had an explanatory power of around 31.31% (Median Absolute Deviance [MAD] = 0.0083, 95% Confidence Interval = [0.30, 0.33]).

To demonstrate stationarity in this and analyses in the and that the MCMC is well-mixed, trace plots for each parameter are plotted in **Figure S1**. Effective numbers of independent samples (N_{eff}) and Gelman-Rubin convergence diagnostic (R) are labelled in each plot. We can see from **Figure S1** that the MCMC is well behaved, as each point is not correlated and is stationary around the mean value. The effective number of uncorrelated points is large for each run, and the convergence statistic is 1.00 (to two decimal places) for all parameters.

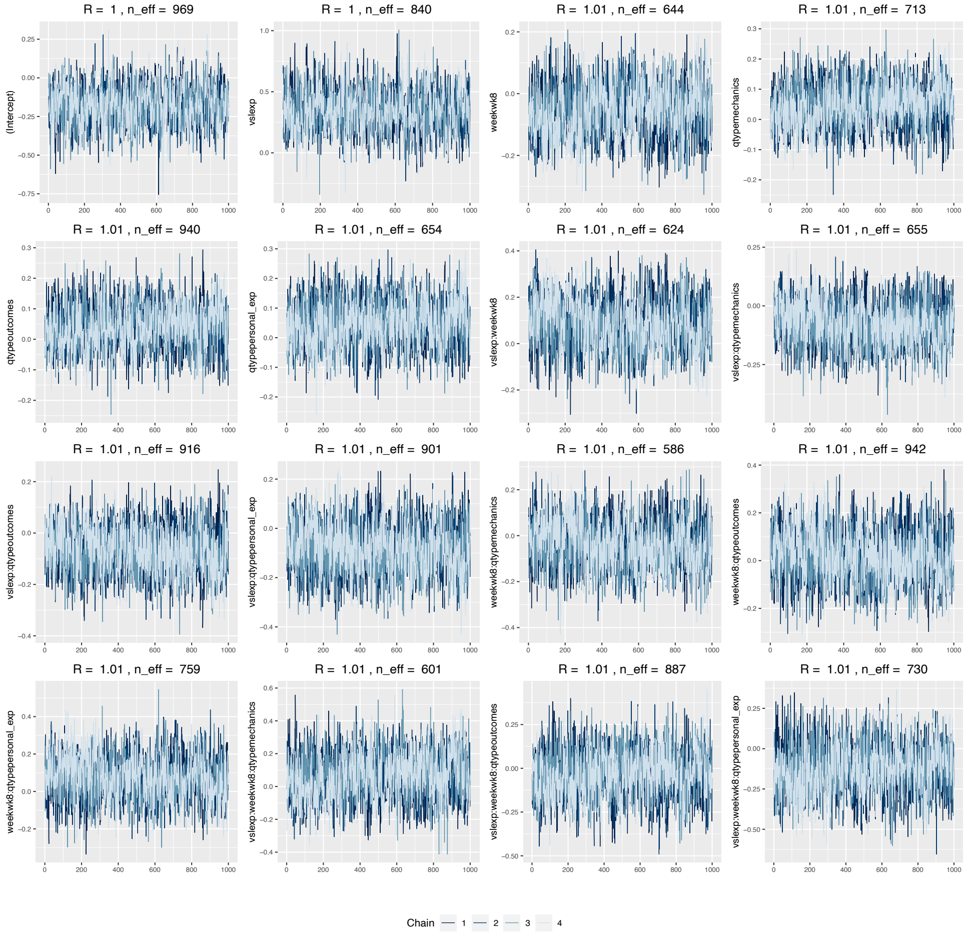

S1 Figure. The trace plots for each parameter in the Bayesian MCMC model, with effective numbers of independent samples (N_{eff}) and Gelman-Rubin convergence diagnostic (R) labelled in each plot.

S2 Table. Model output with Median, Median Absolute Deviance (MAD), 95% Confidence-Interval (CI- CI+), Maximum Probability of Effect (MPE) and Overlap for each term of the model.

Variable	Median	MAD	CI-	CI+	MPE	Overlap
R2	0.31	0.01	0.3	0.33		
(Intercept)	-0.22	0.14	-0.52	0.06		
training	0.35	0.19	-0.04	0.76	95.68	38.16
week	-0.03	0.07	-0.17	0.13	65.18	85.11
Theme (mechanics)	0.1	0.07	-0.02	0.23	93.88	45.05
Theme (outcomes)	0.04	0.06	-0.08	0.17	71.08	78.19
Theme (personal_exp)	0.09	0.07	-0.05	0.23	90.15	52.71
Training * week	0.09	0.09	-0.09	0.28	84.98	61.64
Training * theme (mechanics)	-0.17	0.08	-0.33	0	97.63	30.34
Training * theme (outcomes)	-0.06	0.09	-0.23	0.11	77.18	71.15
Training * theme (personal_exp)	-0.16	0.1	-0.35	0.02	95.33	40.66
week * theme (mechanics)	-0.05	0.09	-0.23	0.13	71.93	77.5
week * theme (outcomes)	0.03	0.09	-0.15	0.22	65.23	85.31
week *: theme (personal_exp)	-0.05	0.1	-0.26	0.15	69.58	80.22
Training * week * theme (mechanics)	0.09	0.12	-0.13	0.32	78.53	70.53
Training * week * theme (outcomes)	-0.06	0.12	-0.29	0.18	67.63	81.91
Training * week * theme (personal_exp)	0.09	0.14	-0.16	0.34	74.95	73.62

Experiment 1. Bayesian model – Utterance results

Our model was specified as

response ~ training * week * valence + (1 | seminar group) + (1 | participant)

The model's used weakly informative priors, set as follows:

~ ~ normal (location = (0, 0, 0, 0, 0, 0, 0), scale = (0.29, 0.29, 0.29, 0.29, 0.29, 0.29, 0.29))

We ran a Bayesian MCMC (link = logit) model (4 chains, each with iter = 2000; warmup = 1000; thin = 1; post-warmup = 1000) using the rstanarm package (v. 2.18). As above, **Figure S2** shows trace plots for each parameter.

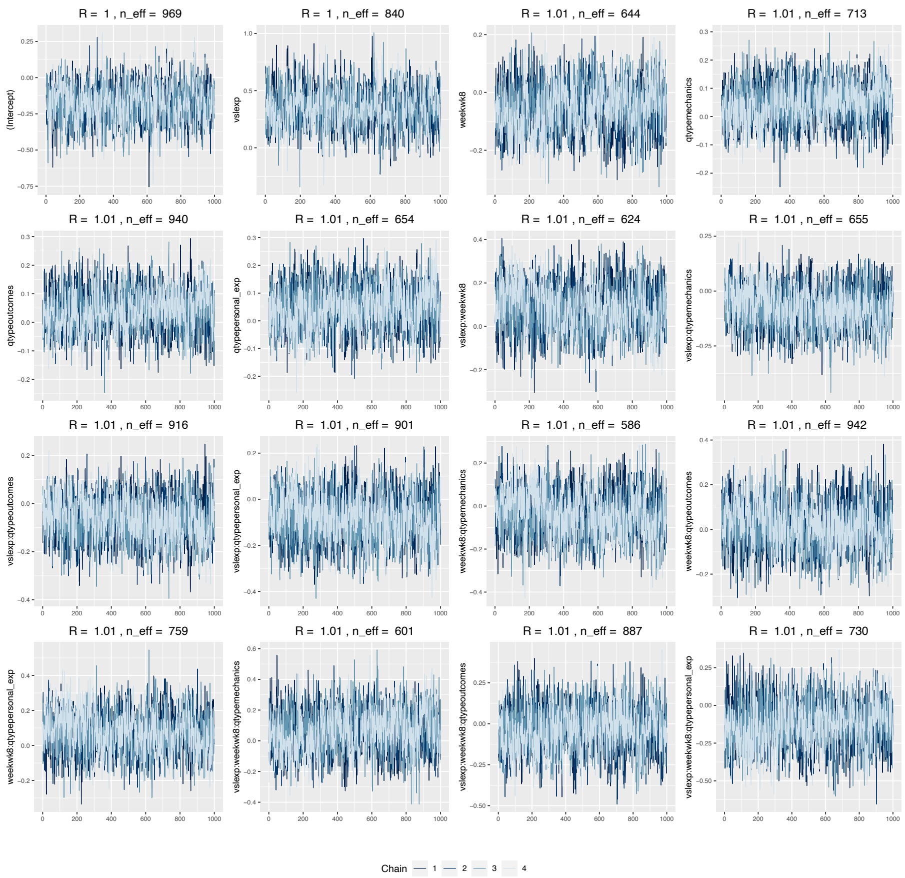

S2 Figure. The trace plots for each parameter in the Bayesian MCMC model, with effective numbers of independent samples (N_{eff}) and Gelman-Rubin convergence diagnostic (R) labelled in each plot.

S3 Table. Model output with Median, Median Absolute Deviance (MAD), 95% Confidence-Interval (CI- CI+), Maximum Probability of Effect (MPE) and Overlap for each term of the model.

**Variable**	**Median**	**MAD**	**CI-**	**CI+**	**MPE**	**Overlap**
R2	0.08	0.03	0.03	0.13		
(Intercept)	0.22	0.02	0.18	0.26		
training	-0.06	0.03	-0.12	-0.01	98.35	29.24
valence	-0.07	0.03	-0.13	-0.02	99.43	21.52
week	-0.01	0.03	-0.06	0.05	62.03	88.23
training * valence	0.14	0.04	0.06	0.22	100	7.06
training * week	0.06	0.04	-0.01	0.14	94.65	43.35
Valence * week	0.02	0.04	-0.06	0.1	70.08	80.07
training * valence * week	-0.12	0.05	-0.22	-0.01	98.8	27.29

Experiment 2. Bayesian model – Survey results

Our model was specified as

response ~ condition * theme + (1 | group) + (1 | participant)

The model's priors were set as follows:

~ normal (location = (0, 0, 0, 0, 0, 0, 0, 0, 0, 0, 0),
scale = (2.49, 2.49, 2.49, 2.49, 2.49, 2.49, 2.49, 2.49, 2.49, 2.49, 2.49))

We ran a Bayesian MCMC (link = logit) model (4 chains, each with iter = 2000; warmup = 1000; thin = 1; post-warmup = 1000) using the rstanarm package (v. 2.18). The model had an explanatory power of around 44.55% (MAD = 0.0095, 95% CI [0.43, 0.46], adj. R2 = 0.42).

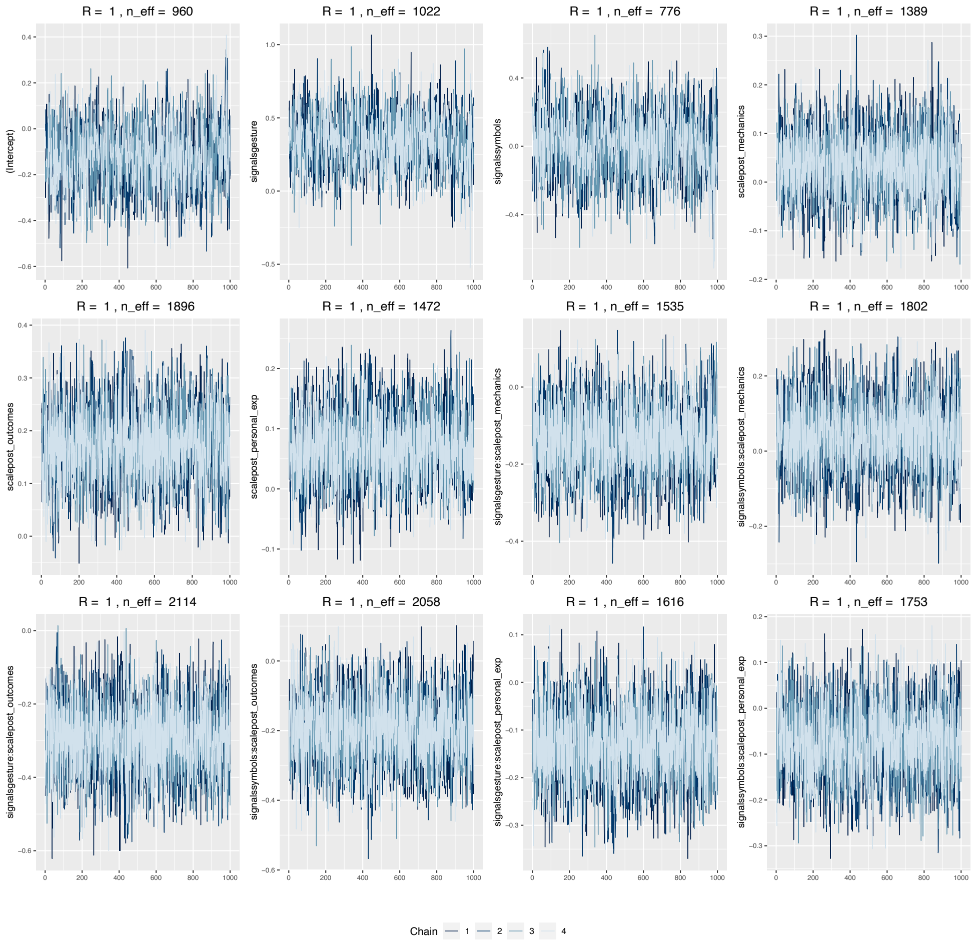

S3 Figure. The trace plots for each parameter in the Bayesian MCMC model, with effective numbers of independent samples (N_{eff}) and Gelman-Rubin convergence diagnostic (R) labelled in each plot.

S4 Table. Model output with Median, Median Absolute Deviance (MAD), 95% Confidence-Interval (CI- CI+), Maximum Probability of Effect (MPE) and Overlap for each term of the model.

Variable	Median	MAD	CI-	CI+	MPE	Overlap
R2	0.45	0.01	0.43	0.46		
(Intercept)	-0.12	0.12	-0.37	0.13		
condition (gesture)	0.33	0.17	0	0.67	97.48	34.18
condition (emoji)	0.01	0.17	-0.36	0.34	52.75	96.67
Theme (mechanics)	0.03	0.06	-0.09	0.16	70.48	79.06
Theme (outcomes)	0.17	0.07	0.03	0.31	99.28	22.35
Theme (personal_exp)	0.07	0.06	-0.04	0.18	90.05	53.64
condition (gesture) * Theme (mechanics)	-0.14	0.09	-0.31	0.04	93.35	44.74
condition (emoji) * Theme (mechanics)	0.04	0.09	-0.14	0.23	67.43	81.62
condition (gesture) * Theme (outcomes)	-0.3	0.09	-0.48	-0.1	99.95	12.78
condition (emoji) * Theme (outcomes)	-0.2	0.1	-0.4	-0.02	97.95	30.81
condition (gesture) * Theme (personal_exp)	-0.13	0.08	-0.28	0.02	95.83	40.43
condition (emoji) * Theme (personal_exp)	-0.08	0.08	-0.21	0.08	83.85	63.03

Experiment 2. Bayesian model – Networked minds results

Our model was specified as

response ~ condition + (1 | group) + (1 | participant)

The model's priors were set as follows:

~ normal (location = (0, 0), scale = (2.50, 2.50))

We ran a Bayesian MCMC (link = logit) model (4 chains, each with iter = 2000; warmup = 1000; thin = 1; post-warmup = 1000) using the rstanarm package (v. 2.18). The model had an explanatory power of around 39.60% (MAD = 0.34, 95% CI [0.062, 0.88], adj. R2 = 0.15).

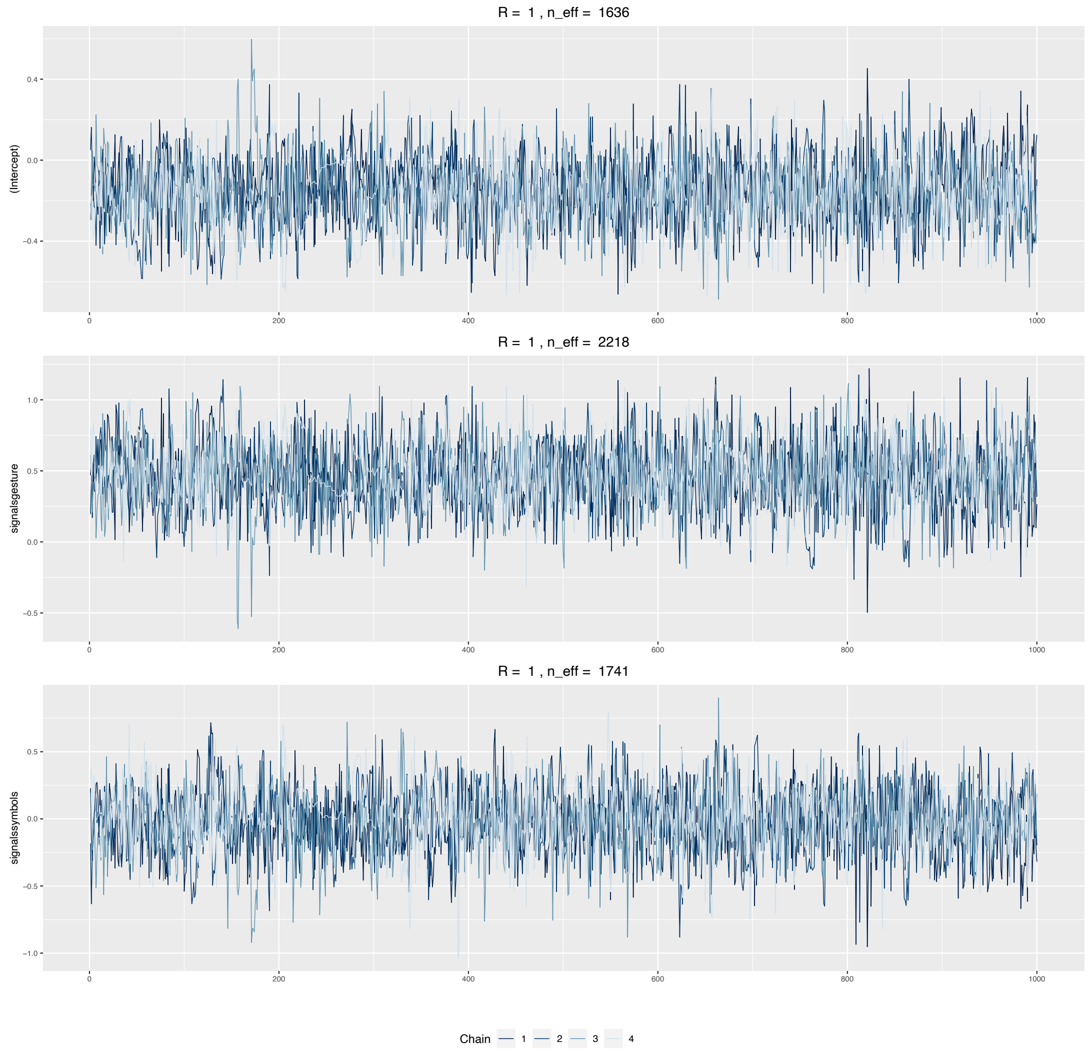

S4 Figure. The trace plots for each parameter in the Bayesian MCMC model, with effective numbers of independent samples (N_{eff}) and Gelman-Rubin convergence diagnostic (R) labelled in each plot.

S5 Table. Model output with Median, Median Absolute Deviance (MAD), 95% Confidence-Interval (CI- CI+), Maximum Probability of Effect (MPE) and Overlap for each term of the model.

Variable	Median	MAD	CI-	CI+	MPE	Overlap
R2	0.4	0.34	0.06	0.88		
(Intercept)	-0.16	0.17	-0.5	0.17		
condition (gesture)	0.46	0.23	0	0.92	97.6	32.43
condition (emoji)	-0.01	0.24	-0.5	0.46	52.13	96.85

Experiment 2. Bayesian model – Similarity of Experience results

Our model was specified as

response ~ condition + (1 | group) + (1 | participant)

The model's priors were set as follows:

~ normal (location = (0, 0), scale = (2.50, 2.50))

We ran a Bayesian MCMC (link = logit) model (4 chains, each with iter = 2000; warmup = 1000; thin = 1; post-warmup = 1000) using the rstanarm package (v. 2.18). The model had an explanatory power of around 50.48% (MAD = 0.45, 95% CI [0.052, 0.96], adj. R2 = 0.17).

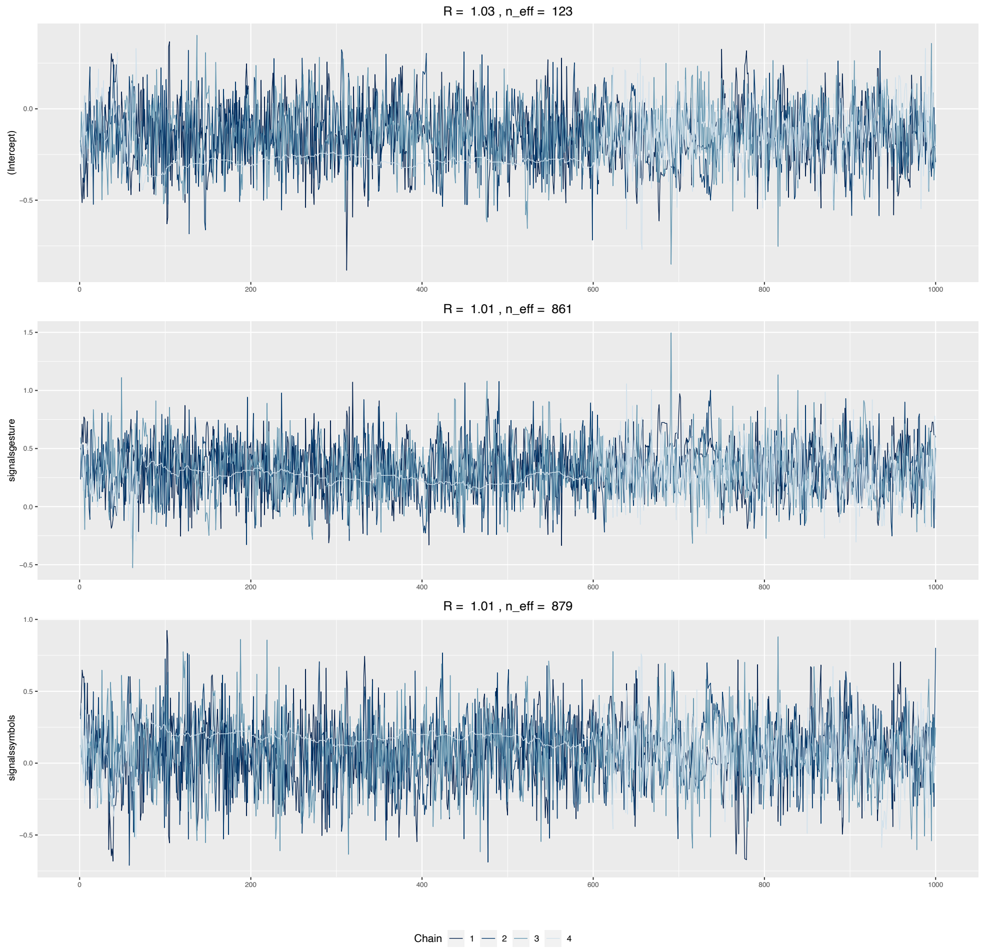

S5 Figure The trace plots for each parameter in the Bayesian MCMC model, with effective numbers of independent samples (N_{eff}) and Gelman-Rubin convergence diagnostic (R) labelled in each plot.

S6 Table. Model output with Median, Median Absolute Deviance (MAD), 95% Confidence-Interval (CI- CI+), Maximum Probability of Effect (MPE) and Overlap for each term of the model.

Variable	Median	MAD	CI-	CI+	MPE	Overlap
R2	0.5	0.45	0.05	0.96		
(Intercept)	-0.17	0.17	-0.47	0.17		
condition (gesture)	0.3	0.2	-0.12	0.75	92.8	46.38
condition (emoji)	0.12	0.19	-0.37	0.53	68.53	79.88

Experiment 2. Bayesian model – IRI results

Our model was specified as

response ~ condition * scale + (1 | group) + (1 | participant)

The model's priors were set as follows:

~ normal (location = (0, 0, 0, 0, 0), scale = (2.50, 2.50, 2.50, 2.50, 2.50))

We ran a Bayesian MCMC (link = logit) model (4 chains, each with iter = 2000; warmup = 1000; thin = 1; post-warmup = 1000) using the rstanarm package (v. 2.18). The model had an explanatory power of around 51.14% (MAD = 0.053, 95% CI [0.40, 0.61], adj. R2 = 0.24)

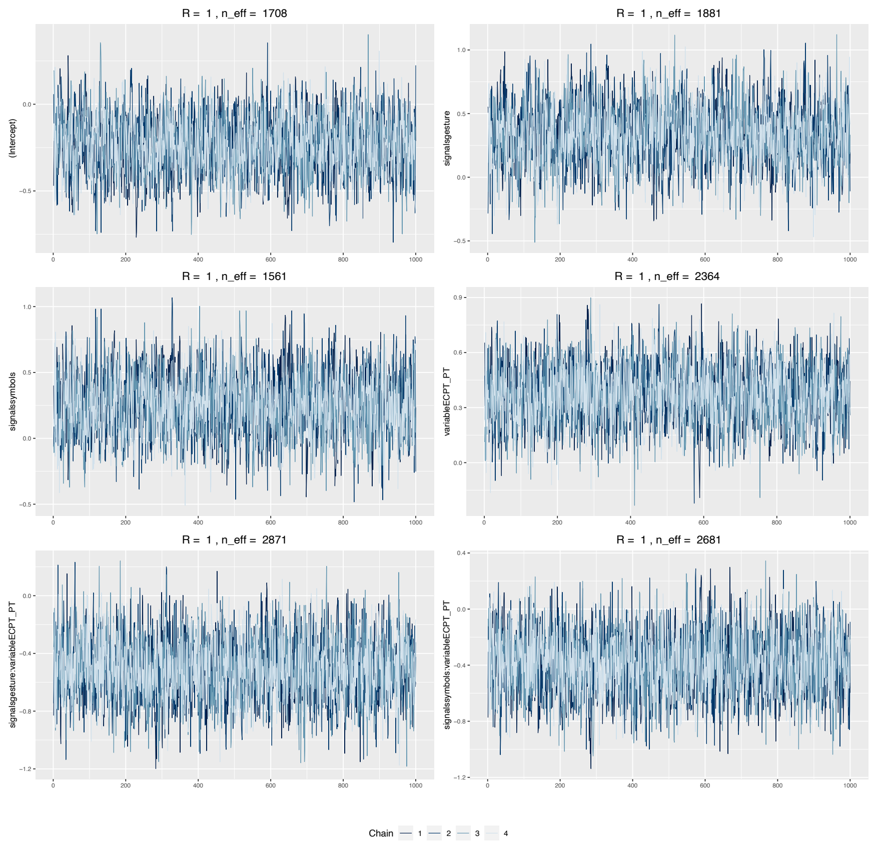

S6 Figure. The trace plots for each parameter in the Bayesian MCMC model, with effective numbers of independent samples (N_{eff}) and Gelman-Rubin convergence diagnostic (R) labelled in each plot.

S7 Table. Model output with Median, Median Absolute Deviance (MAD), 95% Confidence-Interval (CI- CI+), Maximum Probability of Effect (MPE) and Overlap for each term of the model.

**Variable**	**Median**	**MAD**	**CI-**	**CI+**	**MPE**	**Overlap**
R2	0.51	0.05	0.4	0.61		
(Intercept)	-0.24	0.17	-0.57	0.07		
condition (gesture)	0.34	0.23	-0.12	0.81	92.53	46.77
condition (emoji)	0.26	0.24	-0.2	0.7	86.2	59.23
scale (PT)	0.37	0.16	0.06	0.67	98.95	25.16
scale (PT) * condition (gesture)	-0.51	0.22	-0.94	-0.09	99.08	24.64
scale (PT) * condition (emoji)	-0.39	0.23	-0.82	0.03	96.13	38.85

Experiment 2. Bayesian model – transcript results

Our model was specified as

response ~ condition * valence + (1 | group) + (1 | participant)

The model's priors were set as follows:

~ normal (location = (0, 0, 0, 0, 0), scale = (2.50, 2.50, 2.50, 2.50, 2.50))

We ran a Bayesian MCMC (link = logit) model (4 chains, each with iter = 2000; warmup = 1000; thin = 1; post-warmup = 1000) using the rstanarm package (v. 2.18). The model had an explanatory power of around 13.54% (MAD = 0.038, 95% CI [0.063, 0.21]).

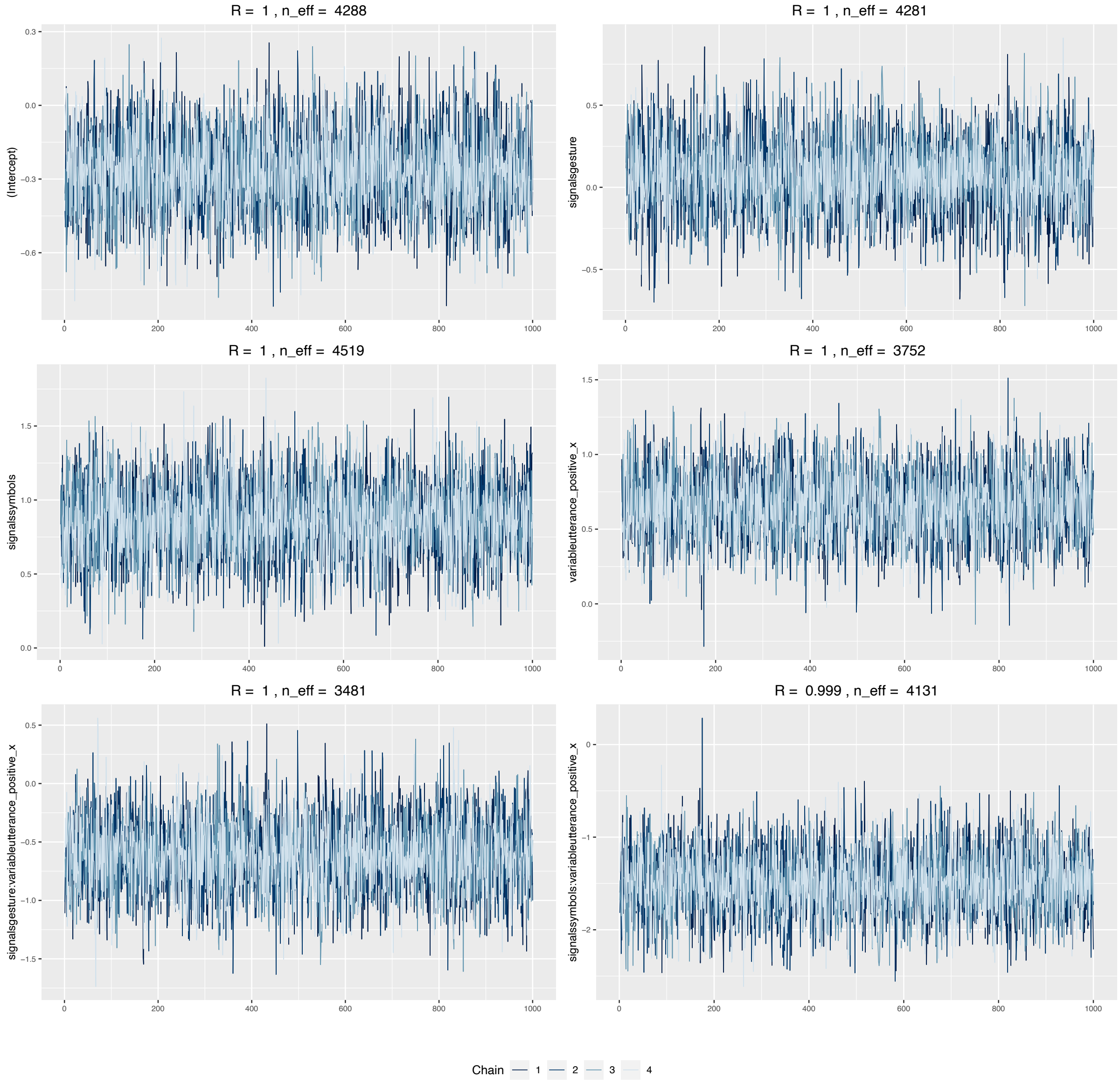

S7 Figure. The trace plots for each parameter in the Bayesian MCMC model, with effective numbers of independent samples (N_{eff}) and Gelman-Rubin convergence diagnostic (R) labelled in each plot.

S8 Table. Model output with Median, Median Absolute Deviance (MAD), 95% Confidence-Interval (CI- CI+), Maximum Probability of Effect (MPE) and Overlap for each term of the model.

Variable	Median	MAD	CI-	CI+	MPE	Overlap
R2	0.14	0.04	0.06	0.21		
(Intercept)	-0.27	0.17	-0.57	0.07		
condition (gesture)	0.07	0.22	-0.38	0.51	62.6	87.49
condition (emoji)	0.86	0.26	0.35	1.36	100	9.63
valence (positive)	0.66	0.22	0.25	1.11	99.78	14.74
valence (positive)* condition (gesture)	-0.61	0.31	-1.22	-0.02	97.73	32.65
valence (positive)* condition (emoji)	-1.49	0.34	-2.18	-0.82	99.98	3.42
