# Supplementary material for: Video meeting signals: Experimental evidence for a technique to improve the experience of video conferencing
Source: PLoS One. 2022 Aug 3;17(8):e0270399. doi: 10.1371/journal.pone.0270399 (PMC9348663; doi:10.1371/journal.pone.0270399)
Supplement: Attachment — Submitted filename: VMS PLOSone RevReply.pdf [file pone.0270399.s002.pdf]

Dear Editor,

Please find below a point by point reply to each comment made in the review process. As you will see, we have now included a second substantial pre-registered experiment, that we believe answer each of the valid concerns that were raised about our first submission.

> Editor Comments
> The manuscript needs some clarifications. More details about how the VMS gestures were selected should be provided to the readers.

As we have now explained in the MS, this set of gestures were developed over time by PDH through working with groups online.

> The theoretical reference model adopted in the design the controlled trial and the criteria adopted for selection of the themes included in the survey (group affiliation, the learning outcomes of that seminar and the mechanics of the seminar interaction) need to be described including the appropriate references.

> The controlled trial design needs to be clarified identifying all trial variables: the manipulated/independent variable (e.g. VMS training or not), controlled variables, dependent variables (e.g. themes included in the survey). The procedure used for construction of scales used for measurement of the themes has to be described mentioning the relevant references.

As we now state in the MS, we used a cluster randomised trial. All trial variables are stated in full in the MS and/or the supplementary material. We specify the statistical models used in our analysis in the main text, and explain in more detail in the supplementary materials. For experiment 1, we could not find a validated scale that was adequate for our use. For this reason, the survey questions were written by us, and grouped into intuitive themes. we were sure to be transparent about our data. In the MS we show the mean responses to individual items, as well as analysing by themes, and the full data set is available online. For the new experiment 2 in our resubmission, we also included two validated measures of social affiliation and interaction.

> Finally, the format of the manuscript has to be reviewed to improve readability and according to the format required by the journal (e. g. order of sections, references).

We have restructured the MS as requested.

> In the ethics statement in the Methods and online submission information, please ensure that you have specified what type you obtained

Consent was given online in the initial survey completed before the first survey. This has been described in the MS.

> We note that Figure 1 includes an image of a participant in the study.

Actually, it does not: that is one of the paper authors (P.H.) who is shown teaching the signals. We have made this clear in the figure legend.

> Reviewer #1:
> Overall, the manuscript was easy to read, clear, and to the point. It seems, based on the results from the data, the VMS system has a positive impact on participant performance. As the authors suggest, they are not able to extrapolate why the VMS system works, but they argue that implementing this type of gestural system in video meetings might be very helpful. I am inclined to agree, but I have a few questions and suggestions for the authors to add to the manuscript to clarify the importance and impact of such a gestural system.

We thank R1 for their kind assessment of the MS and its strengths.

Though the authors show clear differences in the two groups, it is not clear if either group used the "emoticons" and/or the VMS gestures. Is it possible for the authors to descriptively provide this data? Adding the frequency at which emoticons are used in the Zoom session and the rate at which gestures naturally (and post-training) occurred in either setting is also needed to fully understand the impact of the training. Also, did the authors also look at the text-chat during the session and is this included in the lexical analysis?

We are happy to clarify this important point: during the zoom sessions emoticons were not used at all. Indeed, the emoticons that are currently available in zoom (and were employed in our new experiment 2) were not implemented in the zoom platform at the time of the experiment. And so we can be confident they were not used. The text chat was only used in a very limited way to exchange to exchange links and information. In the MS we make these important points clear to the reader.

The finding is simple and preliminary, but I appreciate the authors mention of this, as to not oversell the product. Other than the request for extra detail on the use of the emoticons and gestures, I have no major concerns for the manuscript.

We are delighted with the reviewer's assessment, and hope that the addition of a new, larger experiment that replicates and extends our findings will be welcome.

Reviewer #2:

The reported study addresses a research question of great significance in current times, namely how online/video-communication platforms like Zoom can be improved to allow for more effective and pleasant interactions. There are several things I like about this study, but also some points that give me concern.

We believe that the new experimental data we have now collected and included in this MS convincingly answer the questions that they raise.

Positive points are the number of participants, which is a decent sample size for meaningful analyses of psychometric/social questionnaire measures. Also positive is the use of advanced statistics, including Bayesian techniques. The manuscript is clearly written and easy to read.

We thank the review for this kind assessment of the MS's strengths.

My main point of concern is the apparent lack of a proper control condition. Unless I missed it or misunderstood, it sounds like the control group just received no treatment at all. If this is indeed the case, then any effects measured may be attributable to a pure placebo effect. Such an effect could emerge from the control group's knowledge that they did receive some form of training that was meant to influence interactions, or even simply because they had experienced an additional, in-depth social interaction session for the training. The question is if the control group had received some other treatment that also involved 60 minutes of in-depth social interaction but with a focus on practicing something entirely different, would the same effects have been observed?

Or, even if something different had been taught that was introduced as meaning to benefit interaction (e.g. how to best set the camera angle and sound levels, adjust background etc. to allegedly make interaction most effective), would this have had a comparable effect? Without a proper control treatment, it isn't possible to attribute the effects found to the effectiveness of gesture training per se. Unfortunately, this makes it very difficult to draw meaningful conclusions from this study that are specific to the benefit of the VSM training (over and above any other in-depth group interaction). If the authors disagree I'd be interested to hear their arguments.

We agree completely with the reviewers assessment here. In our discussion we described three key differences between our experimental and control conditions, all of which - including what R2 terms the placebo effect - could be the causal reason for the differences we see.

For this reason, when submitting the MS we pre-registered a set of experiments that could draw more precise conclusions about the benefits of the VSM training. We have now completed one of those studies, and report in the revised manuscript as Experiment 2. We believe this substantially increases the scope and impact of this paper, because we now have strong evidence that precisely addresses the reviewer's only point of concern.

In Experiment 2, new groups of participants had an hour long zoom session. In the VMS condition, they received a 4 minute video instructed them to use of VMS gestures. In the emoji condition, they received a training video that was identical, except that they were told to use zoom reaction buttons to display emojis on their video feeds. In the control condition, they were given no training. As in experiment 1, after the session participants evaluated their interaction and reported their feelings. There is strong evidence that participants in the symbol condition had a worse experience that those in the VMS gesture condition.

We can confidently reject with evidence, therefore, the possibility that R2 raises: that we would get the same benefit with any sort of pre-interaction training. This new experimental contrast allows us to pin the benefits of VMS to the particular use of gesture. When people are trained to signal the same information at the same time with the same training - but to use symbols - there is no advantage. We are confident that R2 will agree that this new data allows for a meaningful conclusion to be drawn about VMS and substantially increases the value of this paper.
