# Supplementary material for: Video meeting signals: Experimental evidence for a technique to improve the experience of video conferencing
Source: PLoS One. 2022 Aug 3;17(8):e0270399. doi: 10.1371/journal.pone.0270399 (PMC9348663; doi:10.1371/journal.pone.0270399)
Supplement: Attachment — Submitted filename: resub 2 Reply.docx [file pone.0270399.s003.docx]

Dear Editor,

Please find below a point by point reply to each comment made in the review of our revised manuscript.

The manuscript improved a lot after revision due to the inclusion of Experiment 2.
Carefully revise the manuscript to correct typos (for instance on page 5 substitute "Statistical analyses:" with "Statistical analyses”).

Done

Reviewer #1: I feel that the authors have more than satisfactorily responded to my comments, and the comments of the other reviewer.

Reviewer #2: I have read the revised manuscript and think the authors have done a great job at addressing the different concerns raised. I am particularly pleased with them including Experiment 2, which indeed does address my main concern.

We are delighted that both reviewers agree that our revisions satisfactorily resolved the comments in their reviews.

1) For experiment 2, rather than referring to the SMs, please include the full model specifications into the statistical analysis section, as was done for Experiment 1. The statistical model applied is a core part of the study and therefore should not be ‘hidden’ in the SMs/more effortful to consult than any of the other details.

We have moved the model specifications from the SM to the main text.

2) One thing I am really missing regarding both experiments is an overview of how much people actually made use of the signals (i.e. gestures and emojis). This is important, because it gives insight into whether the effects are purely down to having the opportunity to draw on the respective signals, or whether actually using them plays a role (I am sure they made good use of them, but the reader should be able to validate this). I don’t expect the authors to add much here in terms of analysis, but summary statistics (e.g., means, sd, min max values) for the different conditions and a comparison between them, minimally, should be included to allow the reader to fully evaluate the findings (especially the difference between the emoji and gesture effects….which in theory could be due to a difference in how much people used the emojis versus the gestures).

Unfortunately, we do not have access to this information in our data. The zoom video recording and transcripts do not code for the use of emojis (or at least, they did not in the version of the software that we employed). Therefore we cannot supply this information to the readers. Crucially, however, this information does not impact the claims that we make in the paper.

Firstly, there might be concern that after the training, participants did not use either gestures or emojis *at all*. In both the gesture and the emoji conditions, participants are trained in their use. The Experimenter asks then to repeat back the signs and practice them, confirming that they are able to use them. And as we discuss in the MS, participants did indeed use the signals as instructed (according to the Experimenter’s anecdotal observations at the time). Most importantly, there are rich differences in participants’ experiences and behaviour between our no training, gesture and emoji conditions, as our data show. So does not appear to be the case that participants ignored or failed to engage with the signalling aspect of the experiment.

Secondly, though they can and did use gestures and emojis, there might be a concern that there was a difference in the frequency of usage between conditions. If participants then choose not to use either the emojis or the gestures, or use one less than the other, then presumably that is because of something intrinsic to those signals: for example, they are less convenient to use, they aren’t as expressive, they don’t help communication. It is precisely those aspects of the use of signals that concerns us, and that we discuss in our results. To repeat: a possible difference in the frequency of usage between conditions does not change the content or evidential strength of any of our claims.

Except for in the headings/titles, the font is too small in figures 2 and 4.

We have used the font size of the headings as the minimum font size throughout. Unfortunately, this meant that we could not include the abbreviated text of each survey item in figure 4. These now refer by index to the full text in the SM.

Typos:
p. 13: Post discussion survey responses were reversed-scored where necessary and normalised within each item.
--> reverse-scored
p. 15 The only difference was that the signal was as emoji rather than a hand gesture.
--> an emoji
p. 15: There were also more positive utterances in the control groups than either of the trainman groups.
--> training?

All these typos have been fixed.
